# Application of Computer-Assisted Endoscopic Ultrasonography Based on Texture Features in Differentiating Gastrointestinal Stromal Tumors from Benign Gastric Mesenchymal Tumors

**DOI:** 10.5152/tjg.2024.22872

**Published:** 2024-05-01

**Authors:** Chengqian Lv, Hongliang Chen, Ping Huang, Yongfang Chen, Bingrong Liu

**Affiliations:** 1 Department of Gastroenterology and Hepatology, The Second Affiliated Hospital of Harbin Medical University, Harbin, China; 2 Department of Gastroenterology and Hepatology, Harbin First Hospital, Harbin, China; 3 Department of Gastroenterology and Hepatology, Hegang Crane Mine Hospital, Hegang, China

**Keywords:** Gastrointestinal stromal tumors, gastric mesenchymal tumors, support vector machine, computer-assisted diagnosis, endoscopic ultrasonography

## Abstract

**Background/Aims:**

Gastrointestinal stromal tumors are common gastric mesenchymal tumors that are potentially malignant. However, endoscopic ultrasonography is poor in diagnosing gastrointestinal stromal tumors. The study investigated the efficacy of texture features extracted from endoscopic ultrasonography images to differentiate gastrointestinal stromal tumors from gastric mesenchymal tumors.

**Materials and Methods:**

The endoscopic ultrasonography examinations of 120 patients with confirmed gastric gastrointestinal stromal tumors, leiomyoma, or schwannoma were evaluated. Histology was considered the gold standard. Three feature combinations were extracted from endoscopic ultrasonography images of each lesion: 48 gray-level co-occurrence matrix-based features, 48 gray-level co-occurrence matrix-based features plus 3 global gray features, and 15 gray-gradient co-occurrence matrix-based features. Support vector machine classifiers were constructed by using feature combinations to diagnose gastric gastrointestinal stromal tumors. The area under the receiver operating characteristic curve, accuracy, sensitivity, and specificity were used to evaluate the diagnostic performance. The support vector machine model’s diagnostic performance was compared with the endoscopists.

**Results:**

The 3 feature combinations had better performance in differentiating gastrointestinal stromal tumors: gray-gradient co-occurrence matrix-based features yielded an area under the receiver operating characteristic curve of 0.90, which was significantly greater than an area under the receiver operating characteristic curve of 0.83 in gray-level co-occurrence matrix-based features and an area under the receiver operating characteristic curve of 0.84 in the texture features plus 3 global features. The support vector machine model (81.67% accuracy, 81.36% sensitivity, and 81.97% specificity) was also better than endoscopists (an average of 69.31% accuracy, 65.54% sensitivity, and 72.95% specificity)

**Conclusion:**

Texture features in computer-assisted endoscopic ultrasonography diagnosis are useful to differentiate gastrointestinal stromal tumors from benign gastric mesenchymal tumors and compare favorably with endoscopists. Support vector machine model using gray-gradient co-occurrence matrix-based texture features revealed the best diagnostic performance in diagnosing gastric gastrointestinal stromal tumors.

**Main Points**

- Endoscopic ultrasonography (EUS) has a poor accuracy in differentiating gastrointestinal stromal tumors (GISTs) from benign GIMTs in the stomach.
- Computer-assisted EUS diagnosis can improve diagnostic accuracy in differentiating gastric GISTs from other benign gastric mesenchymal tumors by using a combination of texture features.
- Among the combination of texture features, the gray-gradient co-occurrence matrix has the highest diagnostic accuracy in diagnosing gastric GISTs.

## Introduction

Gastrointestinal stromal tumors (GISTs) comprise the majority of gastric mesenchymal tumors (GMTs) in the gastrointestinal (GI) tract, accounting for greater than 80% of these lesions.^1^ GISTs may occur in any location of the GI tract but are especially frequent in the stomach (55.6%).^2^ Given the difficulty in predicting the clinical behavior of GISTs, some pathologists consider that all GISTs should be practically regarded as potentially malignant.^1,2^ In general, GISTs in the stomach show a better prognosis after surgical resection than GISTs in the small intestine.^2-4^ Therefore, it is crucial to differentiate gastric GISTs from other benign gastric GIMTs, such as schwannomas and leiomyomas.^5^

Endoscopic ultrasonography (EUS) is valuable for diagnosing and evaluating gastric GISTs. Endoscopic ultrasonography imaging can provide detailed images of the GI wall structure and indicate the layer of origin of the lesion, enabling differential diagnosis.^6-8^ However, EUS imaging alone has poor accuracy for diagnosing gastric subepithelial tumors, including GISTs and leiomyomas.^9,10^ Furthermore, the diagnostic accuracy of EUS images alone depends on the endoscopists’ experience, and no consensus exists regarding EUS imaging characteristics, such as the heterogeneity of the echo.^11,12^ Although EUS-guided fine-needle aspiration (EUS-FNA) can obtain the tissue of GIMTs for pathological examination and is more accurate for diagnosing specific types of GIMTs,^13^ the procedure is invasive and expensive. Therefore, it is necessary to explore a noninvasive and more accurate approach to differentiate GISTs from benign GIMTs in the stomach. As an objective and noninvasive tool to improve diagnostic accuracy, computer-assisted diagnosis (CAD) has recently been widely employed in the field of medical diagnosis for the differentiation of numerous pathologic lesions,^14^ such as breast tumor detection.^15,16^ Indeed, previous reports have encouraged the application of CAD in EUS.^17-19^

In this paper, we aimed to develop and validate a computer-assisted EUS diagnosis based on texture features distinguishing GISTs from benign mesenchymal tumors in the stomach, exploring an objective and noninvasive and superior approach to EUS compared to EUS diagnosis based on endoscopists.

## MATERIALS and Methods

### Patients

All patients considered in this study underwent an EUS examination at the Endoscopic Unit of the Second Affiliated Hospital of Harbin Medical University from October 2012 to May 2019. The lesions were confirmed as gastric GISTs, schwannomas, or leiomyomas by postoperative histopathology. Endoscopic ultrasonography reports and histopathologic reports obtained from the patient’s online medical records were reviewed.

### Acquisition of EUS Images

All EUS examinations were performed by 2 endoscopists using an ultrasound catheter probe (GF-UM2R; Olympus, Tokyo, Japan), a radial-scanning ultrasonic endoscope (GF-UM260; Olympus), and an EUS processor (EU-ME1; Olympus). At least 10 EUS images obtained for each lesion were saved digitally in Windows bitmap format. Some lesions were excluded due to the poor quality of EUS images, which was caused by unclean gastric juice or gas interference. Endoscopic ultrasonography images were reviewed by experienced endoscopists who were blinded to the final diagnosis. Finally, one still EUS image of high quality was selected for each of the remaining lesions for further digital image analysis.

### Histopathology

The tumors were classified into GISTs or leiomyomas and schwannomas by immunohistochemical pathology.^20^ The specific immunohistochemical profile of GISTs was c-kit (CD117), CD34, or DOG-1-positive. Leiomyomas were desmin-positive and c-kit-negative, while schwannomas were S-100-positive and c-kit-negative. All GIMTs were classified into the GIST group and non-GIST (schwannoma and leiomyoma) groups based on potential malignancy.

### Digital Image Analysis

**Figure 1.**
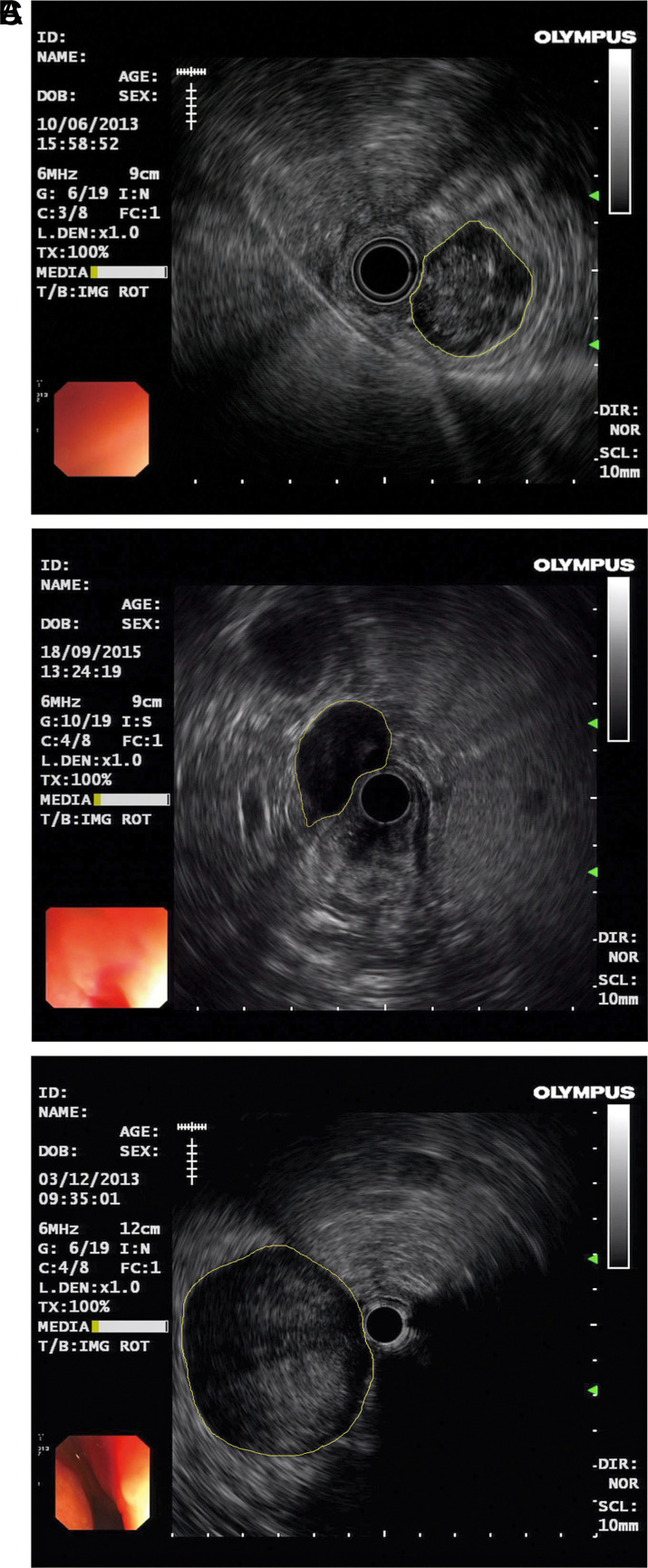
Illustration of ROIs in EUS images. (A) GIST, (B) leiomyoma, (C) schwannoma. ROIs were marked with yellow lines. EUS, endoscopic ultrasonography; GIST, gastrointestinal stromal tumor; ROIs, regions of interest.

An experienced endoscopist annotated the lesion areas (regions of interest, ROIs) of all EUS images used in this paper, i.e., the region surrounded by the yellow line shown in Figure 1. Three examples from the GIST, leiomyoma, and schwannoma groups are presented in Figure 1A-C, respectively. For all ROIs, we extracted texture features to build classification models. In this study, we employed 3 feature combinations: 48 gray-level co-occurrence matrix (GLCM)-based features, 48 GLCM-based features plus 3 global gray features,^21^ and 15 gray-gradient co-occurrence matrix (GGCM)-based features.^22^

### The First Feature Combination

For each ROI, 3 distances (*d* = 1, 2, 3) and 4 directions (*θ* = 0°, 45°, 90°, 135°) were used to compute the co-occurrence matrix. From the co-occurrence matrix, 4 descriptors were calculated: contrast, correlation, energy, and homogeneity. Thus, we obtained 3 × 4 × 4 = 48 GLCM-based features.

### The Second Feature Combination

This combination included 48 GLCM-based features and 3 global gray features based on the entire GLCM. The 3 features included entropy, mean, and SD.

### The Third Feature Combination

In this paper, the gray level was 256. We computed the gradient matrix from the gray matrix using the 3 × 3 window Sobel operator and normalized the gradient matrix to 32 levels. Then, we computed the GGCM *H*, where each element *H*(*i*, *j*) represented the count of pixels whose gray values and gradient values were *i* and *j*, respectively. Fifteen texture features were extracted from the GGCM: little gradient dominance, large gradient dominance, gradient heterogeneity, gray heterogeneity, energy, gradient average, gray average, gradient mean square error, gray mean square error, correlation, gray entropy, hybrid entropy, inertia, and inverse difference moment.

### Classification Method

**Figure 2.**
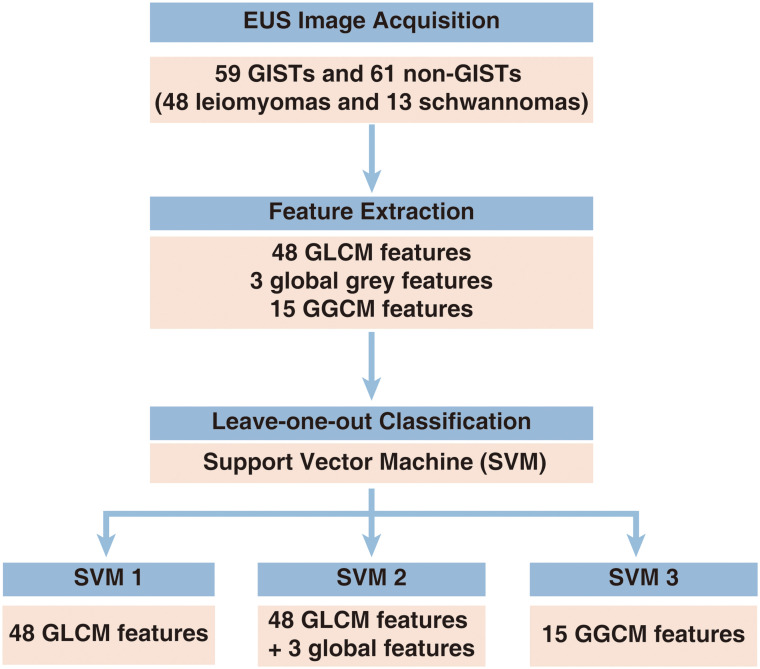
Architecture of computer-assisted detection system.

In this study, we used a support vector machine (SVM) model to differentiate GISTs from non-GISTs (leiomyoma and schwannoma). Support vector machine classifiers were constructed using these 3 feature combinations to recognize gastric GISTs. We applied the leave-one-out cross-validation method, whereby at each time, one sample was chosen for testing, and the remaining samples were used for training; the process was repeated n times (where n is the sample size). The experiment was performed using MATLAB R2016a (MathWorks Inc., Natick, Massachusetts, USA), and the default parameters in SVM were applied. The structure is shown in Figure 2. Second, we evaluated the diagnostic accuracy of 3 experienced and 3 junior endoscopists in differentiating gastric GISTs from non-GISTs. The presumptive diagnostic accuracy was determined by comparing the endoscopists’ impressions with histologic findings.

### Performance Evaluation

We denoted GISTs as the positive class and non-GISTs as the negative class. We evaluated the classification performance using sensitivity, specificity, and accuracy. The area under the receiver operating characteristic curve (AUC) was used to assess the discrimination of SVM models based on 3 feature combinations. The performance was compared between the CAD system and 6 endoscopists. Experienced endoscopists were considered to have performed more than 600 EUS examinations of GIMTs, and junior endoscopists performed fewer than 300 EUS examinations.

### Statistical Analysis

The clinical information of patients, such as patient age and lesion size, was described as the mean ± SD. Categorical variables are described as frequencies and percentages. The differences between the clinical information of the 2 groups of patients were compared with the *t*-test and chi-square test. The differences between the different feature combinations were compared based on AUC with Delong’s test. The differences in the classification results between the CAD system and those endoscopists were compared with the chi-square test. A *P* value less than .05 was considered statistically significant. All data were analyzed using Statistical Package for Social Sciences 17.0 (SPSS, Inc., Chicago, Ill, USA) and SigmaPlot 13.0 (Systat Software Inc., San Jose, California, USA).

### Ethics Committee Approval

All patients provided written informed consent to participate. The study protocol was reviewed and approved by the Ethics Committee of the Second Affiliated Hospital of Harbin Medical University (Ethics review batch number: KY2022-272).

## Results

A total of 120 patient EUS images were included in the study, in which 59 GISTs (49.17%) were regarded as the positive class, and 61 non-GISTs (50.83%) were regarded as the negative class, including 48 leiomyomas and 13 schwannomas. The patients’ clinical characteristics are presented in Table 1. All tumors were hypoechoic and originated from the muscularis propria. Compared with the non-GIST group, the GIST group had the following characteristics: older average age, larger tumor diameter, and a higher proportion of male patients. The details are also in Table 1. The presumptive diagnostic accuracy of 3 experienced and 3 junior endoscopists in differentiating gastric GISTs from non-GISTs (leiomyomas and schwannomas) is presented in Table 2. Interestingly, there was no significant difference in the diagnostic accuracy between the 3 experienced and 3 junior endoscopists.

**Figure 3.**
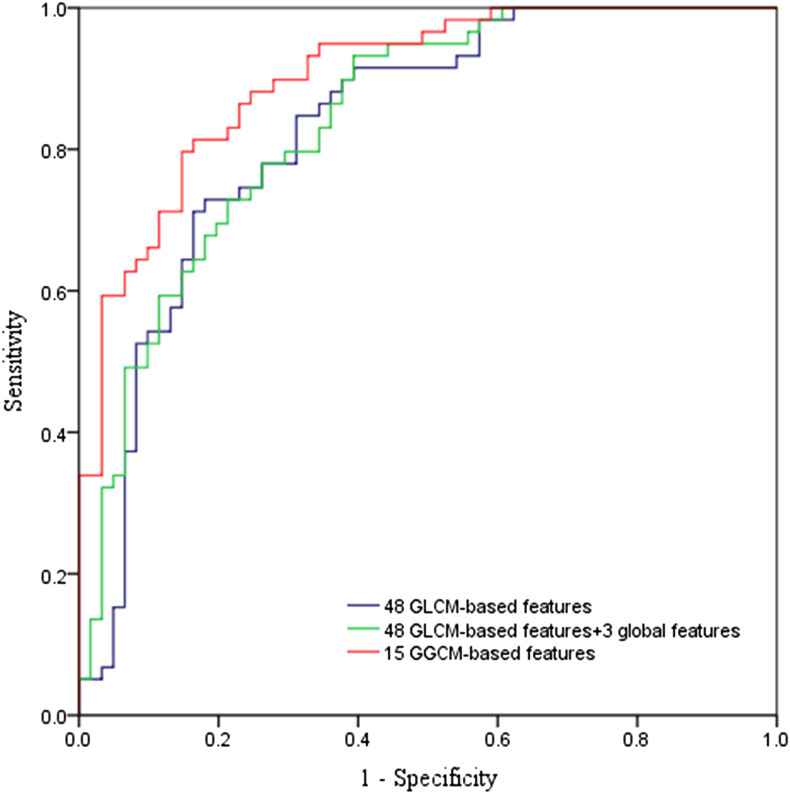
The receiver operator characteristic curves obtained using 3 feature combinations.

The classification results obtained by SVM using the 3 feature combinations are presented in Table 3. The receiver operator characteristic curves are shown in Figure 3. The statistical results regarding AUC values, 95% CIs, and *P* values are shown in Table 4. The AUC obtained using 15 GGCM-based texture features (0.90) was significantly larger than that using 48 GLCM-based texture features (0.83, *P* < .01) and that using 48 GLCM-based texture features plus 3 global features (0.84, *P* < .05). The classification performance based on the texture analysis of the GGCM was superior to the other methods in differentiating GISTs from non-GISTs. The sensitivity of the classification result obtained by SVM using 15 GGCM-based texture features was 81.36%, the specificity was 81.97%, and the accuracy was 81.67%. Its diagnostic accuracy was significantly higher than that of experienced and junior endoscopists, and the details are shown in Table 5.

## Discussion

In this study, we developed a new computer-assisted EUS diagnosis system to differentiate gastrointestinal stromal tumors from benign mesenchymal tumors of the stomach using texture features. Support vector machine modeling using GGCM-based texture features had high accuracy in diagnosing gastric GIMTs, which was superior to the other methods in differentiating GISTs from non-GISTs. To the best of our knowledge, this is the first study to report on using SVM modeling based on GGCM texture features to differentiate gastric GIMTs.

In our study, we obtained some similar findings as in previous studies. The average age of the GIST patients was approximately 58 years old, and GISTs were slightly more prevalent in men than in women. Gastrointestinal stromal tumors have a larger tumor diameter.^23-25^

Previous studies have shown that EUS imaging alone has poor accuracy and is insufficient to accurately diagnose third- (submucosa) and fourth-layer (muscularis propria) hypoechoic masses, including GISTs and leiomyomas.^9,10^ Therefore, in this study, all the mesenchymal tumors we selected were hypoechoic and originated from the muscularis propria. We found that the most incorrect EUS diagnoses occurred with leiomyomas and schwannomas, which were misdiagnosed as GISTs. This result is similar to those of previous studies^9,10^ because the diagnostic accuracy of EUS imaging alone is related to the endoscopists’ subjective experience.^25^ However, in our study, what surprised us is that the diagnostic ability between the experienced endoscopists and the junior endoscopists was not significantly different. Therefore, extensive training and practice may not improve endoscopists’ abilities. In recent years, some procedures for acquiring more tissue, such as EUS-guided fine-needle biopsy (EUS-FNB) and mucosal incision-assisted biopsy, have been recommended to differentiate gastric GIMTs,^1^ but all of these procedures are invasive. Therefore, CAD will be a beneficial and noninvasive method to improve diagnostic accuracy.

Endoscopic ultrasonography images are composed of pixels, which are basic finite elements of the digital image; the arrangement and combination of these pixels can reflect the texture and structure of the tumors in EUS images.^26^ The subtle differences among different tumors can be represented in images, although these changes are often beyond the perceptive ability of visual interpretation. Therefore, computers can distinguish GISTs from non-GISTs according to features extracted from EUS images, which is more scientific than the naked eye and has improved accuracy. However, there is still no consensus regarding EUS imaging characteristics among endoscopists.^11,12^ Fortunately, analysis by computer techniques that are objective and comprehensive can capture such subtle differences.^14^ CAD can compute and analyze relevant mathematical parameters using the technologies of image processing and image understanding. Previous studies have reported that CAD with EUS is helpful in diagnosing gastrointestinal submucosal tumors and gastric GIMTs.^19, 27-28^

Thus, some objective information from EUS images can be extracted using computer techniques to reflect the potential differences in all types of tumors, and CAD was applied as an objective tool in our study. We directly extracted GLCM-based features and GGCM-based features from the EUS images without preprocessing the images. In contrast to previous studies, all EUS images used in this study were selected to obtain high imaging quality and clear image characteristics; thus, the frequency of echoendoscopy was not unified. This feature is consistent with the operation in practice. In fact, our study showed that this is feasible. The SVM classification method was built using 3 feature combinations to recognize gastric GISTs. Our results showed that GGCM-based features performed significantly better than GLCM-based features. The gray matrix reflects the brightness and contrast of EUS images and denotes the echogenicity information, whereas the gradient matrix reflects the variation in brightness and contrast and denotes the degree of heterogeneity. Given that we did not normalize the obtained EUS images, the brightness values may be influenced by the raw images. Therefore, the gray matrix may not accurately reflect the difference between gastric GISTs and other benign GIMTs. Given that the gradient matrix may be less influenced by unstandardized images, GGCM-based features obtained better results. Thus, we conclude that the gradient matrix can better reflect the EUS features of gastric GISTs.

Gray-gradient co-occurrence matrix-based features extracted from EUS images are superior. The SVM model using GGCM-based texture features showed an accuracy of 81.67%, a sensitivity of 81.36%, and a specificity of 81.97%. In addition, GGCM-based features yielded the largest AUC (0.90). This significantly increased compared with GLCM-based features or GLCM-based texture features plus 3 global features. Its diagnostic performance was significantly higher than that of endoscopists.

We assessed the classification performance by calculating the AUCs, which were defined as the statistical measures of the predictive power, and it accounts for the dependency of specificity on sensitivity and is independent of specific cut-off values. The AUCs of SVMs using 3 feature combinations in this study were greater than 0.80, demonstrating their strong predictive abilities. Specifically, GGCM-based features yielded the largest AUC (0.90).

In our study, we used a leave-one-out cross-validation method for training and testing. For training, all examples but one were employed to train the SVM model. Then, the remaining example was tested using the SVM classifier to predict whether the lesion was a gastric GIST. This process was repeated 120 times until every example was classified as the test example. The SVM classification method also has the advantage of dealing with small sample sets with good generalization performance. Support vector machine has been used for medical diagnoses, such as EUS and Holter electrocardiograms.^28,29^ SVM typically shows good generalized performance, which contributes to structural risk minimization.^30^

In recent years, computer-assisted diagnosis with the convolutional neural network (CNN-CAD) system has also been applied to the differential diagnosis of GMTs, which is also encouraging.^31-33^ The CNN-CAD system does not need to extract the EUS features separately and is more intelligent and more convenient. However, its internal structure is complex, and it is similar to a “black box.” We cannot understand the texture extraction process and cannot explain which texture features were extracted. The CNN-CAD system needs large-scale EUS images for training, but the image data of GMTs are relatively small, which limits the advantages of the convolutional neural network model. Therefore, in our study, SVM modeling using texture features was applied to distinguish GISTs from non-GIST tumors. This method is suitable for small sample sets, and the process of texture feature extraction and classification can be explained and easily understood. These 2 methods have their own advantages and disadvantages. In addition, we have collected an increasing number of EUS images to develop a more suitable CAD system for GMTs, which may be an SVM model using texture features, CNN-CAD, or a combination of the 2 methods.

At present, this CAD of gastric mesenchymal tumors has not yet been applied in making clinical decisions in the real world. For GMTs, National Comprehensive Cancer Network (NCCN) guidelines recommend surgical resection for those >5 cm, EUS-FNA/B or mucosal incision-assisted biopsy to diagnose GISTs from non-GIST tumors between 2 cm and 5 cm, and follow-up by endoscopy or EUS once or twice a year for tumors <2 cm in size without high-risk EUS features.^34,35^ However, GISTs cannot be simply considered benign or malignant tumors by either clinical investigations or invasive pathological examinations. Thus, the other guidelines recommend surgical resection when a submucosal tumor is diagnosed as GIST, even if <2 cm.^36,37^ To the best of our knowledge, CAD may be useful in choosing the next diagnosis or treatment modality for GISTs. Therefore, we further attempted to validate the effectiveness of CAD for preoperative diagnosis in GISTs <2 cm in size, alone or combined with an invasive pathological examination in GISTs ≥2 cm in size.

This study still has limitations. First, the sample size is relatively small. Second, although our study showed that texture features are useful for distinguishing GISTs from benign GIMTs in the stomach, further study should be conducted with regard to applying multi-frame images to extract texture features, as using several EUS images of different views will provide more texture information, and the diagnosis accuracy may be improved. In the future, we hope to develop general methods for diagnosing gastric GISTs that are accurate and can be widely applied.

Texture features in computer-assisted EUS diagnosis are useful for differentiating gastrointestinal stromal tumors from benign mesenchymal tumors in the stomach, which may be complementary to EUS diagnosis of GISTs in clinical practice. The classification performance of computer-assisted EUS diagnosis is better than that of endoscopists. Furthermore, GGCM-based features extracted from EUS images are more suitable for building SVM to recognize GISTs, and further study is warranted.

## Figures and Tables

**Table 1.** Clinical Characteristics of the Patients

Characteristics	GISTs	Non-GISTs	
Number of cases	59	48 leiomyomas	13 schwannomas
Age (years)	26-75 (58.02 ± 10.03)	21-69 (50.39 ± 10.00)^*^	
Sex (male)	31 (52.54%)	14 (22.95%)^*^	
Size (cm, maximum diameter)	0.7-11 (3.17 ± 2.05)	0.6-6 (2 ± 1.30)^*^	
Lesion location, n (%)			
Cardia	1 (1.69)	22 (45.83)	0 (0)
Fundus	23 (38.98)	19 (39.58)	1 (7.69)
Body	22 (37.29)	6 (12.5)	11 (84.62)
Antrum	13 (22.03)	1 (2.08)	1 (7.69)

^*^A significant difference between the 2 groups, *P* < .05.

GIST, gastrointestinal stromal tumor; non-GIST, nongastrointestinal stromal tumor, including leiomyoma and schwannoma; EUS, endoscopic ultrasonography.

**Table 2.** Presumptive Diagnosis by 3 Experienced and 3 Junior Endoscopists in Differentiating Gastric GISTs from Non-GISTs

Endoscopists	TP	TN	Sensitivity (%)	Specificity (%)	Accuracy (%)
Experienced 1	38	46	64.41	75.41	70.00
Experienced 2	39	46	66.1	75.41	70.83
Experienced 3	40	43	67.80	70.49	69.17
Junior 1	40	42	67.80	68.85	68.33
Junior 2	37	46	62.71	75.41	69.17
Junior 3	38	44	64.41	72.13	68.33

EUS, endoscopic ultrasonography; GIMTs, gastrointestinal mesenchymal tumors; GIST, gastrointestinal stromal tumor; non-GIST, nongastrointestinal stromal tumor; TN, true negative; TP, true positive.

**Table 3.** Accuracy of the 3 Feature Combinations

Classification Method	TP	TN	Sensitivity (%)	Specificity (%)	Accuracy (%)
48 GLCM-based features	43	48	72.88	78.69	75.83
48 GLCM-based features + 3 global features	44	46	74.58	75.41	75.00
15 GGCM-based features	48	50	81.36	81.97	81.67

EUS, endoscopic ultrasonography; GGCM, gray-gradient co-occurrence matrix; GLCM, gray-level co-occurrence matrix; TN, true negative; TP, true positive.

**Table 4.** AUC, 95% CI, and *P* Values of 3 Feature Combinations

Feature Combination	AUC	95% CI	*P*
48 GLCM-based features	0.83^*^	[0.76, 0.90]	.0066
48 GLCM-based features + 3 global features	0.84^*^	[0.77, 0.91]	.0199
15 GGCM-based features	0.90	[0.85, 0.95]	–

^*^Significant differences compared with 15 GGCM-based features using DeLong’s test (*P* < .05).

AUC, the area under the receiver operating characteristic; GGCM, gray-gradient co-occurrence matrix; GLCM, gray-level co-occurrence matrix.

**Table 5.** Diagnostic Performance of Computer-Assisted Detection System Compared to That of Endoscopists in Differentiating Gastrointestinal Stromal Tumors from Non-GISTs

Evaluation Indexes	15 GGCM-Based Features	Experienced 1	Experienced 2	Experienced 3	Junior 1	Junior 2	Junior 3
Sensitivity (%)	81.36	64.41^*^	66.10	67.80	67.80	62.71^*^	64.41^*^
Specificity (%)	81.97	75.41	75.41	70.49	68.85	75.41	72.13
Accuracy (%)	81.67	70.00^*^	70.83^*^	69.17^*^	68.33^*^	69.17^*^	68.33^*^

^*^Significant differences compared with the CAD system (*P* < .05). CAD, computer-assisted detection system; GISTs, gastrointestinal stromal tumors.
